# Biogenesis, Function, and Applications of Virus-Derived Small RNAs in Plants

**DOI:** 10.3389/fmicb.2015.01237

**Published:** 2015-11-09

**Authors:** Chao Zhang, Zujian Wu, Yi Li, Jianguo Wu

**Affiliations:** ^1^Key Laboratory of Plant Virology of Fujian Province, Institute of Plant Virology, Fujian Agriculture and Forestry UniversityFuzhou, China; ^2^Peking-Yale Joint Center for Plant Molecular Genetics and Agrobiotechnology, The National Laboratory of Protein Engineering and Plant Genetic Engineering, College of Life Sciences, Peking UniversityBeijing, China

**Keywords:** RNA silencing, small RNAs, virus-derived small RNAs, DCLs, RDRs, AGOs, plant antiviral defense

## Abstract

RNA silencing, an evolutionarily conserved and sequence-specific gene-inactivation system, has a pivotal role in antiviral defense in most eukaryotic organisms. In plants, a class of exogenous small RNAs (sRNAs) originating from the infecting virus called virus-derived small interfering RNAs (vsiRNAs) are predominantly responsible for RNA silencing-mediated antiviral immunity. Nowadays, the process of vsiRNA formation and the role of vsiRNAs in plant viral defense have been revealed through deep sequencing of sRNAs and diverse genetic analysis. The biogenesis of vsiRNAs is analogous to that of endogenous sRNAs, which require diverse essential components including dicer-like (DCL), argonaute (AGO), and RNA-dependent RNA polymerase (RDR) proteins. vsiRNAs trigger antiviral defense through post-transcriptional gene silencing (PTGS) or transcriptional gene silencing (TGS) of viral RNA, and they hijack the host RNA silencing system to target complementary host transcripts. Additionally, several applications that take advantage of the current knowledge of vsiRNAs research are being used, such as breeding antiviral plants through genetic engineering technology, reconstructing of viral genomes, and surveying viral ecology and populations. Here, we will provide an overview of vsiRNA pathways, with a primary focus on the advances in vsiRNA biogenesis and function, and discuss their potential applications as well as the future challenges in vsiRNAs research.

## Introduction

In plants and other eukaryotic organisms, small RNAs (sRNAs) have emerged as key players of RNA silencing in the regulation of various biological processes, including plant growth and development, host immunity and pathogen virulence. To date, a series of pathways beginning with different precursors and resulting in sRNAs of various sizes with dedicated functions has been elucidated in the model plant *Arabidopsis* through both forward and reverse genetic screens.

Small RNAs are grouped into two major classes: microRNAs (miRNAs) and small interfering RNAs (siRNAs). MiRNAs are generated from a primary transcript (pri-miRNA) containing a stem–loop structure and processed into 20–25 nucleotide (nt) sRNAs by RNase III endonuclease DICER-LIKE 1 (DCL1), with the exception of the DCL4-dependent miRNAs such as miR822, miR839, and miR859 (Rajagopalan et al., 2006; Ben Amor et al., 2009). Many other cellular proteins are also required for miRNAs metabolism from processing to degradation, such as Methyltransferase HUA ENHANCER1 (HEN1), zinc finger protein SERRATE (SE), dsRNA-binding protein HYPONASTIC LEAVES 1 (HYL1), G-patch domain-containing protein TOUGH (TGH), HEN1 SUPPRESSOR1 (HESO1) and a family of SMALL-RNA DEGRADING NUCLEASES (SDNs) reviewed in Xie et al. (2015). siRNAs are divided into four subgroups: *trans*-acting siRNAs (ta-siRNAs), heterochromatic-associated siRNAs (hc-siRNAs), natural antisense transcript siRNAs (nat-siRNAs), and virus-activated siRNAs (vasiRNAs), which are primarily generated from diverse perfectly double-stranded RNA precursors and excised by DCL1 and its homologs (DCL2, DCL3, and DCL4) either cooperatively or redundantly (Bouche et al., 2006; Axtell, 2013; Cao et al., 2014). sRNAs are recruited by an ARGONAUTE (AGO) ribonucleoprotein complex, referred to as the RNA-induced silencing complex (RISC; Poulsen et al., 2013; Carbonell and Carrington, 2015). The programmed sRNA/RISC targets and silences RNA or DNA through post-transcriptional gene silencing (PTGS) or transcriptional gene silencing (TGS), respectively, in a sequence-specific manner (Martinez de Alba et al., 2013). Several updated reviews regarding the functions of miRNAs and siRNAs in controlling a plenty of biological processes are available (Harfouche et al., 2015; Weiberg and Jin, 2015; Zhang, 2015).

In regard to virus-derived small RNAs (vsiRNAs), many studies have shown in various organisms that the production of vsiRNAs is linked to antiviral immunity via silencing the viral genomic RNA. Two lines of evidences, including the biogenesis of vsiRNAs and the viral suppressors of RNA silencing (VSRs)-regulated counter-defense help us to understand the vsiRNAs-mediated antiviral RNA silencing pathway. In this review, we mainly summarize the current knowledge of the biogenesis and function of vsiRNAs in plants and discuss their potential applications as well as their future challenges in crop breeding systems.

## Biogenesis of Virus-Derived Small RNAs in Plants

Virus-derived siRNAs, a class of exogenous sRNAs, are one of the earliest discovered sRNAs (Hamilton and Baulcombe, 1999). The biogenesis of vsiRNAs, however, has not been clearly elucidated over the past decade. What is known has been deduced based on the insights from genetic analysis in *Arabidopsis* and vsiRNAs profiling from various plant species. It indicates that the biogenesis of vsiRNAs is analogous to that of endogenous sRNAs, which requires the actions of various DCLs, RDRs, and AGOs. **Table 1** lists the specific plant DCLs, RDRs, and AGOs known to contribute to the biogenesis of vsiRNAs.

**Table 1:** Plant DCLs, RDRs, and AGOs involve in the vsiRNA biogenesis and function.

Virus	Genus	Host	DCLs	RDRs	AGOs	Reference
TRV	*Tobravirus*	*Arabidopsis thaliana*	DCL2/3/4	RDR1/2/6	AGO2/4	Fusaro et al., 2006; Donaire et al., 2008; Ma et al., 2015
TuMV	*Potyvirus*	*Arabidopsis thaliana*	DCL2/4	RDR1/2/6	AGO1/2/5/7/10	Garcia-Ruiz et al., 2010, 2015; Carbonell et al., 2012
TMV	*Tobamovirus*	*Arabidopsis thaliana*	Unknown	RDR1/6	Unknown	Qi et al., 2009
ToRSV	*Nepovirus*	*Nicotiana benthamiana*	Unknown	Unknown	AGO1	Ghoshal and Sanfaçon, 2014
CMV	*Cucumovirus*	*Arabidopsis thaliana*	DCL1/2/3/4	RDR1/6	AGO1/2/4/5	Bouche et al., 2006; Diaz-Pendon et al., 2007; Wang et al., 2010, 2011
BMV	*Bromovirus*	*Arabidopsis thaliana*	DCL2/4	RDR6	AGO1	Dzianott et al., 2012
TCV	*Carmovirus*	*Arabidopsis thaliana*	DCL2/4	Unknown	AGO1/2/7	Qu et al., 2008; Harvey et al., 2011; Zhang et al., 2012
TBSV	*Tombusvirus*	*Nicotiana benthamiana*	Unknown	Unknown	AGO2	Scholthof et al., 2011; Schuck et al., 2013
PVX	*Potexvirus*	*Arabidopsis thaliana, Nicotiana benthamiana*	DCL2/4	RDR6	AGO2/5	Schwach et al., 2005; Bouche et al., 2006; Andika et al., 2015; Brosseau and Moffett, 2015
TYLCV	*Begomovirus*	*Nicotiana benthamiana, cassava*	DCL2/3	Unknown	Unknown	Akbergenov et al., 2006
CaMV	*Caulimovirus*	*Arabidopsis thaliana*	DCL1/2/3/4	Unknown	AGO4	Blevins et al., 2006; Raja et al., 2014
BCTV	*Curtovirus*	*Arabidopsis thaliana*	DCL3	Unknown	AGO4	Raja et al., 2008
CaLCuV	*Begomovirus*	*Arabidopsis thaliana*	DCL1/2/3/4	RDR1/2/6-independent	AGO4	Blevins et al., 2006; Aregger et al., 2012; Raja et al., 2014
RSV	*Tenuivirus*	*Oryza sativa*	Unknown	RDR6	AGO1/18	Jiang et al., 2012; Wu et al., 2015
RDV	*Phytoreovirus*	*Oryza sativa*	Unknown	RDR6	AGO1/18	Hong et al., 2015; Wu et al., 2015
OsEV	*Endornavirus*	*Oryza sativa*	DCL2	Unknown	Unknown	Urayama et al., 2010

*Specific plant DCLs, RDRs, and AGOs known to contribute to the biogenesis of vsiRNAs. TRV, *Tobacco rattle virus*; TuMV, *Turnip mosaic virus*; TMV, *Tobacco mosaic virus*; ToRSV, *Tomato ring spot nepovirus*; CMV, *Cucumber mosaic virus*; BMV, *Brome mosaic virus*; TCV, *Turnip crinkle virus*; PVX, *Potato virus X*; TYLCV, *Tomato yellow leaf curl virus*; CaMV, Cauliflower *mosaic virus*; BCTV, *Beet curly top virus*; CaLCuV, Cabbage *leaf curl virus*; RSV, Rice *stripe virus*; RDV, *Rice dwarf virus*; OsEV, *Oryza sativa endornavirus*.*

### Origin of Viral siRNAs

Early models of the origin of vsiRNAs hypothesized that the double-stranded replication intermediates (RIs) of positive-strand RNA viruses trigger the activation of the production of vsiRNAs (Ahlquist, 2002). If this was the case, the read counts of vsiRNAs derived from both the positive and negative strand in the virus-infected host plant cells should be similar. However, profiling of vsiRNAs derived from *Cymbidium ringspot virus* (CymRSV)-infected plants led to several interesting observations: (i) vsiRNAs derived from the genomic or positive strand far outnumbered those derived from the negative strand; (ii) most vsiRNAs were concentrated in a limited number of hotspots in the CymRSV genome; and (iii) double-stranded vsiRNAs bound to VSR protein p19 appeared to contain mismatches, as they were more sensitive to RNase A digestion than fully double-stranded controls (Ahlquist, 2002). These observations and others suggest that vsiRNAs are derived from the processing of highly structured regions of genomic RNA rather than from the perfectly paired dsRNAs-like RIs (Molnar et al., 2005; Koukiekolo et al., 2009). Several recent vsiRNA profiling studies in diverse eukaryotes have revealed that there are hotspots for vsiRNAs generation and there is a clear preference for a polarity, which have similar vsiRNA distribution to CymRSV (Xu et al., 2012; Vives et al., 2013; Visser et al., 2014). These results show, at least for some viruses, that RIs do not contribute significantly to the biogenesis of vsiRNAs. Rather, dsRNA-like secondary structures of single-stranded (ss) viral RNAs are most likely the dominant source of vsiRNAs (Donaire et al., 2009; Szittya et al., 2010; Wang et al., 2010). There is limited research on the origin of vsiRNAs derived from DNA viruses. This was characterized in *geminiviruses*, a family of plant DNA viruses with one or two circular ssDNA genomes that are replicated via dsDNA intermediates by a rolling circle mechanism. The dsRNA-like structures of DNA viruses are dominantly formed by the annealing of converging sense/antisense transcripts (Chellappan et al., 2004; Aregger et al., 2012).

### Roles of DCLs in the Biogenesis of Viral siRNAs

**FIGURE 1:**
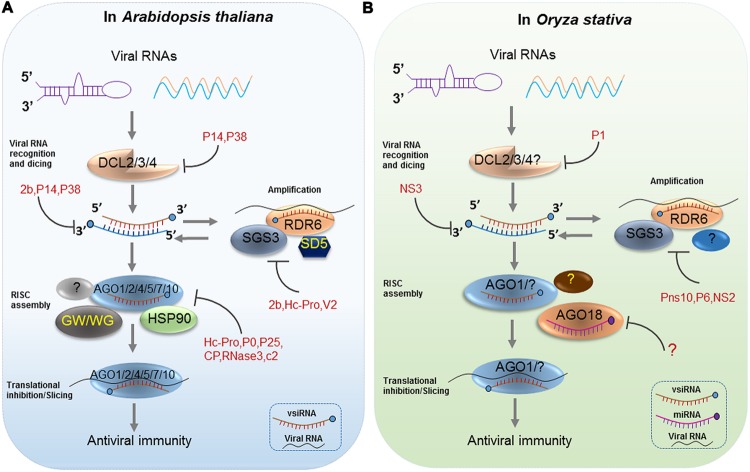
**Antiviral RNA Silencing in *Arabidopsis* and *Oryza stativa*.** RNA silencing is initiated by the recognition of viral dsRNAs or partially double-stranded hairpin RNAs, which are processed into viral siRNAs (vsiRNAs) by Dicer-like proteins (DCL2/3/4) in *Arabidopsis* **(A)** and *Oryza stativa* **(B)**. Next, HSP90-activated AGO1/2/4/5/7/10 is loaded with a vsiRNA, thereby forming a large RNA-induced silencing complex (RISC), which has likely incorporated other proteins such as the GW motif-containing AGO interactors in *Arabidopsis* **(A)**. In rice, AGO18 positively regulates AGO1 binding to vsiRNAs by sequestering miR168 **(B)**. Afterward, the vsiRNA-loaded RISC targets viral RNAs by slicing or translational inhibition. Secondary vsiRNAs are produced in an amplification loop through the actions of RNA-dependent RNA polymerases (RDRs) and cofactors (SGS3 and SDE5). Diverse viral silencing suppressors (VSRs) target various steps of antiviral silencing in plants **(A)** and **(B)**.

Members of the plant Dicer-like (DCL) protein family are the critical components of the RNA-silencing pathway that produce vsiRNAs of different lengths (DCL4: 21-nt, DCL2: 22-nt, DCL3: 24-nt; Deleris et al., 2006). In *Arabidopsis*, the production of siRNAs from plant RNA viruses is mainly catalyzed under hierarchical action of the enzyme activities of DCL4 and DCL2 (Deleris et al., 2006; **Figure 1**). These results were first verified with *Tobacco rattle virus* (TRV) infected *dcl* combination mutants. Specifically, TRV-specific siRNAs accumulated as discrete 21- and 24-nt species in wild-type (WT) *Arabidopsis*, similar to that in *dcl2* mutants, while in *dcl3*, and *dcl2*/*dcl3* double mutants, only 21-nt siRNAs accumulated, suggesting that DCL4 is mainly responsible for the processing of 21-nt vsiRNAs from RNA viruses (Fusaro et al., 2006; Garcia-Ruiz et al., 2010). In the case when DCL4 is absent or its activity is reduced or inhibited by virus, DCL2 produces 22-nt vsiRNAs. Although the abundance is lower, they are sufficient to trigger protective immunity, thus rescuing antiviral silencing. In the *dcl2*/*dcl4* double mutant, DCL3 can produce 24-nt vsiRNAs, which play a minor role in antiviral defense. Additionally, Bouche et al. (2006) reported the DCLs functions in the production of *Cucumber mosaic virus* (CMV)-specific siRNA. Their results indicate that CMV siRNAs are produced by DCL4 in WT plants, or DCL2 and DCL3 in *dcl4* mutants, which behave similar to ta-siRNAs. They also detected very low levels of 21-nt CMV-siRNAs in *dcl2/dcl3/dcl4* triple mutant plants, suggesting that DCL1 could produce 21-nt CMV-siRNAs in the absence of DCL2, DCL3 and DCL4, although much less efficiently than ta-siRNAs (Bouche et al., 2006). Likewise, this group reported an opposite situation of DCLs activities in the production of Turnip crinkle virus (TCV)-specific siRNAs in *Arabidopsis*. They found that DCL2 was the major contributor of TCV siRNAs, but DCL4 could also produce TCV siRNAs in the absence of DCL2 (Bouche et al., 2006). They later gave an interpretation for the discrepancy of DCL-dependent vsiRNAs processing between TCV and CMV. They thought DCL2 produced TCV siRNAs dominantly because DCL4 was inhibited by TCV but not because DCL2 had a stronger affinity than DCL4 for TCV dsRNA. In the *Brome mosaic virus* (BMV)-inoculated *Arabidopsis* Columbia-0 (Col-0) plants, BMV RNA accumulated higher level than that in *dcl2*/*dcl4* double mutant plants but similar to that in *dcl2* or *dcl4* single mutant plants (Dzianott et al., 2012). As for the silencing suppressor (HC-Pro)-deficient *Turnip mosaic virus* (TuMV)-infected *Arabidopsis*, blot assays showed that the 21-nt vsiRNA from the 5′ UTR region were sensitive to loss-of-function in DCL4, but in the absence of DCL4, this region yielded siRNAs that were 22-nt in length and dependent on DCL2 (Garcia-Ruiz et al., 2010). Recently, the differential requirement for DCL4 and DCL2 proteins in the inhibition of intracellular and systemic infection by *Potato virus X* (PVX) in *Arabidopsis* was also reported (Andika et al., 2015). In rice, there are six putative DCL proteins (OsDCL1, OsDCL2a, OsDCL2b, OsDCL3a, OsDCL3b, and OsDCL4). Liu et al. (2007) found that OsDCL4 is the major Dicer responsible for the generation of 21-nt siRNAs that are associated with inverted repeat transgenes and ta-siRNAs derived from the endogenous TAS3 gene and OsDCL1, but not OsDCL4, is important for miRNA accumulation. However, of the six rice DCLs, their function in vsiRNA biogenesis remain poorly understood. Interestingly, rice DCL2 negatively affected maintenance of *Oryza sativa endornavirus* (OsEV), an endogenous dsRNA virus by increasing the accumulation of vsiRNAs, and indicated that rice DCL2 plays a major role in processing vsiRNA from OsEV (Urayama et al., 2010). Functions of rice DCLs in vsiRNAs biogenesis are always deduced from their homology to *Arabidopsis* DCLs. However, Xu et al. (2012) found that the distribution of vsiRNAs derived from *Rice stripe virus* (RSV), a negative-strand RNA virus, is very different in rice, *Nicotiana benthamiana*, and *Laodelphax striatellus* (insect vector). This suggests that the origins of vsiRNAs derived from different hosts are likely distinct.

Although the biogenesis of vsiRNAs by DCLs has been extensively documented in plant RNA viruses, little is known regarding viruses with DNA genomes. Indeed, the vsiRNAs biogenesis pathway of plant DNA viruses, which have a dsDNA intermediate stage, differs from that of RNA viruses (Chellappan et al., 2004; Akbergenov et al., 2006; Blevins et al., 2006). For instance, *Geminiviruses* trigger accumulation of various vsiRNA classes. Specifically, in *begomoviru*s-infected *N. benthamiana* and *cassava*, three major size-classes of vsiRNAs, 21-, 22-, and 24-nt, are generated, and more than one DCL is involved in their biogenesis (Akbergenov et al., 2006). DCL3 is required to produce 24-nt siRNAs, while the production of the 22-nt siRNAs depends on DCL2, as well as other as yet unidentified DCL activities. From the studies of another *geminivirus, Cabbage leaf curl virus* (CaLCuV), DCL1 also processes some CaLCuV long dsRNAs into residual 21-nt vsiRNAs in the absence of DCL2, DCL3, and DCL4 (Blevins et al., 2006). In the case of plant viruses with a dsDNA genome, such as *Cauliflower mosaic virus* (CaMV), 21-, 22-, and 24-nt siRNAs from both sense and antisense polarities, coding and non-coding regions accumulated in CaMV-infected *Arabidopsis* (Blevins et al., 2006). DCL3 and DCL2 are required for the biogenesis of 24- and 22-nt CaMV siRNAs, respectively. Furthermore, the key role of DCL1 in the biogenesis 21-nt siRNAs from CaMV leader region was confirmed by the finding that high levels of 21-nt CaMV siRNAs accumulated in *dcl2/dcl3/dcl4*. Taken together, unlike siRNA derived from RNA viruses, current knowledge suggests the accumulation of vsiRNAs from DNA viruses requires the action of all four DCL enzymes in a coordinated and hierarchical manner.

### Roles of RDRs and AGOs in the Biogenesis of Viral siRNAs

Mechanistically, the biogenesis of viral siRNAs consists of two phases: DCL-dependent initiation phase and RDR-dependent amplification phase. RDRs play important roles in a second round of vsiRNA formation following viral RNA replication- or high-structural dsRNA-triggered biogenesis of primary siRNAs. The process of secondary siRNA-generation is thought to be similar to the RDR6-dependent tasi-RNA pathway. Specifically, primary vsiRNAs initiate the PTGS process involving diverse functional AGOs with RNase H-type activity (discussed latter) in plants. PTGS pathways recruit suppressor of gene silencing 3 (SGS3, a protein that blocks RNAs degradation), a putative RNA trafficking protein SDE5, and the RDR6 to transform cleaved ssRNA fragments into long, perfect dsRNA precursors. Then, these are subsequently processed into 21-nt siRNAs by DCL4 and its dsRNA binding partner DRB4 or 22-nt siRNAs by DCL2 (Dalmay et al., 2000; Mourrain et al., 2000; Gasciolli et al., 2005; Bouche et al., 2006; Martinez de Alba et al., 2015). Thus, it is assumed that an amplification loop is established whereby siRNA-guided AGO1 cleavage of viral transcripts leads to the RDR6/SGS3/SDE5-dependent production of dsRNAs, which serves as a substrate for the DCL-dependent formation of secondary vsiRNAs (Voinnet, 2008; Mallory and Vaucheret, 2009, 2010; **Figure 1**). In the beginning, there was some indirect evidence to support the role of host RDRs in vsiRNAs biogenesis and antiviral defense. First, host RDRs’ activity is induced after virus infection. For instance, in tobacco mosaic virus (TMV)-infected tobacco leaves, a host polymerase RDR activity was stimulated by TMV (Romaine and Zaitlin, 1978). Moreover, loss-of-function mutations in host RDRs lead to hyper-susceptibility to virus infection (Dorssers et al., 1982; Xie et al., 2001; Schwach et al., 2005). Later, profiles of vsiRNAs in RDR mutants by deep sequencing directly revealed that host RDRs play a role in vsiRNAs biogenesis (Donaire et al., 2008; Wang et al., 2010). Among the six RDR genes that are encoded by the *Arabidopsis* genome, the functions of RDR1, RDR2, and RDR6 have been well demonstrated. The production of CMV-derived siRNAs in the absence of the CMV VSR protein 2b is largely dependent on RDR1 originally (Diaz-Pendon et al., 2007). Wang et al. (2010) found that there is a twofold decrease in the relative abundance of viral siRNAs in CMV-infected *rdr1*/*rdr6* double and *rdr1/rdr2/rdr6* triple mutants plants compared to *rdr1* and WT plants, suggesting RDR1 and RDR6 are all required for amplification of CMV-derived siRNAs. They also suggested that RDR1 preferentially amplifies the 5′-terminal end of the three genomic RNAs of CMV, whereas RDR6 amplifies the remaining regions (Wang et al., 2010, 2011). Consistent with these studies, another group found that both RDR1 and RDR6 play important roles in biogenesis of TMV-Cg siRNAs in virus-infected cells (Qi et al., 2009). The biogenesis of TRV- and TuMV-derived siRNAs involves the combined activity of RDR1, RDR2, and RDR6 (Donaire et al., 2008; Garcia-Ruiz et al., 2010). In rice, there are five RDR genes that have been annotated (Zong et al., 2009) but no insights to their roles in the biogenesis of vsiRNAs was provided to date except for the findings given by Jiang et al. (2012). They were the first to report that rice RDR6 plays a role in the accumulation of siRNAs derived from RSV genomic RNA. Through deep sequencing, they found that compared to the WT, the abundance of the RSV-derived siRNAs showed a 53% reduction in OsRDR6 knockdown transgenic plants in which transcription levels were extremely reduced (Jiang et al., 2012). Recently, the same group confirmed the function of RDR6 in *Rice dwarf virus* (RDV) vsiRNA biogenesis. They found that the accumulation of RDV vsiRNAs was reduced in the OsRDR6 knockdown transgenic plants (Hong et al., 2015).

The biogenesis of RDR-dependent secondary vsiRNAs requires activity of AGOs, which serve as the effector proteins functioning in the antiviral RNA silencing. In *Arabidopsis*, there are 10 AGOs, categorized into three clades, while rice has 19 AGOs classified into four clades (Morel et al., 2002; Nonomura et al., 2007; Vaucheret, 2008). Many reports have shown that *Arabidopsis* AGO1, AGO2, AGO5, and AGO10 act in PTGS targeting RNA viruses (Qu et al., 2008; Harvey et al., 2011; Scholthof et al., 2011; Carbonell et al., 2012; Ghoshal and Sanfaçon, 2014; Brosseau and Moffett, 2015; Carbonell and Carrington, 2015; Garcia-Ruiz et al., 2015; Ma et al., 2015). For rice, little is known regarding how the various AGOs regulate antiviral RNA silencing except for AGO1 and AGO18, which synergistically play a role in antiviral defense (**Figure 1**; Du et al., 2011a; Wu et al., 2015). Actually, the role of AGOs in vsiRNAs biogenesis is almost restricted to their effector activity in the PTGS pathway. Specifically, AGOs associate with vsiRNAs to target complementary viral RNAs, and the cleaved ssRNAs are source of production of RDR-dependent secondary vsiRNAs.

### Suppression of Viral siRNAs Biogenesis by Various VSRs

In the arms race between hosts and viruses, viruses developed potent VSRs that can target multiple steps of RNA silencing pathway to counter host antiviral strategies (Csorba et al., 2015; Wieczorek and Obrepalska-Steplowska, 2015). Apparently, the hallmark of the antiviral silencing response is the DCL-dependent production of vsiRNAs, thus the biogenesis of vsiRNAs is one important target of various VSRs. VSRs have ability to block the vsiRNA biogenesis by inhibiting DCL proteins and/or co-factors activity, sequestrating dsRNA/siRNA or AGO protein destabilization prior of RISC assembly. For instance, in the presence of TCV P38, siRNAs are undetectable, suggesting P38 acts to suppress DCL’s activity (Qu et al., 2003). Deleris et al. (2006) later demonstrated using genetic analysis that P38 inhibits DCL4 but not DCL2. Moreover, P1 of RYMV also has ability to inhibit DCLs activities including both DCL4 and DCL3 (Lacombe et al., 2010; Weinheimer et al., 2010; Gillet et al., 2013). Some VSRs, such as *Pothos latent aureusvirus* (PolV) P14, TCV p38, CMV 2b, and RSV NS3 have been described to bind dsRNAs in a size-independent manner and therefore to block vsiRNA maturation (Merai et al., 2005; Deleris et al., 2006; Goto et al., 2007; Xiong et al., 2009). Furthermore, other VSRs such as RDV Pns10, CMV 2b, and *Rice yellow stunt virus* (RYSV) P6 block secondary siRNA biogenesis by downregulating RDR6 expression or suppressing the activity of RDR1, which are critical for secondary siRNAs synthesis (Diaz-Pendon et al., 2007; Ren et al., 2010; Guo et al., 2013). RSV NS2 as well as V2 protein of *Tomato yellow leaf curl virus* (TYLCV) directly interact with SGS3, the cofactor of RDR6, and compete with SGS3 for dsRNA binding (Glick et al., 2008; Fukunaga and Doudna, 2009; Kumakura et al., 2009; Du et al., 2011b; **Figure 1**). *Potyvirus* HC-Pro, one of the best characterized viral silencing suppressors, plays multiple roles in the suppression of vsiRNAs biogenesis, such as ds-siRNA binding, blocking HEN1 methyltransferase, HEN1 binding, blocking primary siRNA biogenesis by RAV2 interaction, RDR6 downregulation. A comprehensive review on the current knowledge of the diverse VSRs and their strategies to suppress vsiRNAs biogenes is available (Csorba et al., 2015).

Taken together, these findings support a model for vsiRNA biogenesis in plants in which DCL4 processes various viral dsRNA transcripts into primary vsiRNAs; RDRs and AGOs produce secondary vsiRNA through amplification. However, VSRs can inhibit the production of vsiRNAs by countering multiple steps of the antiviral silencing pathway. Notably, questions such as how, when, and where in the cell vsiRNAs are initially accessed by the RNA silencing machinery remain elusive.

## Roles of Virus-Derived Small RNAs in Antiviral Defense

Besides the cellular components involved in vsiRNA biogenesis, there is accumulating evidence that the vsiRNA themselves are directly playing roles in antiviral immunity in plants. Considering the general function of sRNAs in directing RISC to target transcripts for PTGS or TGS, it is easy to assume that the main role of vsiRNAs is to target viral mRNA molecules. This may be a more effective method to inhibit viral replication than the production of primary vsiRNAs by DCL-dependent action on the RIs or highly structural dsRNA derived from the viral genome.

### VsiRNAs Function to Downregulate Viral RNAs

Virus-derived small interfering RNAs are recruited by diverse AGOs to form RISC and direct the degradation of viral RNA molecules through PTGS in a sequence-specific manner (Simon-Mateo and Garcia, 2006). AsatsiR-12, an artificial siRNA derived from the satellite RNA (satRNA) of SD-CMV (a severe field Shan-Dong strain), which targets the 3′ UTR of CMV RNA and triggers the RDR6-dependent antiviral silencing pathway, has a positive effect on antiviral defense. The accumulation of CMV RNAs was reduced in the CMV-Δ2b-infected transgenic plants expressing asatsiR-12 but this reduction was inhibited by the 2b suppressor (Zhu et al., 2011). Their findings provided the first demonstration that viral satRNAs could mediate silencing against their helper virus. However, whether any AGO protein is involved in the silencing process as part of an active vsiRNA-RISC was not demonstrated at that time. Later, plenty of *in vitro* experiments involving different combinations of cell-free systems and viruses were conducted and results have shown the existence of endonucleolytic of antiviral RISCs but not directly AGOs (Szittya and Burgyan, 2013). The slicer activity of AGOs was verified by using *in vitro* assays involving cytoplasmic extracts of evacuolated tobacco protoplasts (Iki et al., 2010; Schuck et al., 2013). For instance, ‘RISC formation/cleavage assay’ and ‘replication inhibition assay’ showed that AGO1, AGO2, AGO3, AGO5, AGO7 and AGO10 had slicer activity with 21- and 22-nt siRNAs and thus inhibited RNA replication of TBSV *in vitro* (Schuck et al., 2013). Actually, *in vivo* experiments have indicated that *N. benthamiana* AGO with similarity to *Arabidopsis* AGO2 is involved in antiviral defense against TBSV (Scholthof et al., 2011; Odokonyero et al., 2015). A recent study on the antiviral role of AGO2 has shown that AGO2, AGO10, and to a lesser extent AGO1 associate with vsiRNAs derived from HC-Pro-deficient TuMV-AS9 (Garcia-Ruiz et al., 2015). Until now, *Arabidopsis* AGO1, AGO2, AGO5, AGO7, AGO10, and rice AGO1 and AGO18 are all the AGOs reported that associate with vsiRNAs upon virus infection, reviewed in Carbonell and Carrington (2015; **Figure 1**). Notably, previous studies on miRNA-mediated antiviral RNA silencing have revealed that many molecular chaperones, such as HSP90 and GW/WG motif-containing proteins, assist in or promote the antiviral defense response (Mayor et al., 2007; Iki et al., 2010, 2012; Earley and Poethig, 2011; Yang et al., 2012). Here, we hypothesized that the vsiRNA-mediated antiviral RNA silencing is likely to require various molecular chaperones (**Figure 1**). However, to date, no evidence verifies this. Additionally, there is still much that remains missing from our knowledge of vsiRNA-mediated antiviral defense due to several obstacles: (i) It remains unknown whether the majority of, if not all, vsiRNAs can load into specific AGO proteins; (ii) Even though some AGO proteins play a role in virus infection and are able to bind vsiRNAs, the RISC complex alone may not be able to access target the viral genome due to their complex secondary structures or VSR activity; (iii) At present, evidence that specific vsiRNAs can target the viral genome for inhibition of virus infection *in vivo* is limited. Therefore, the efficiency of antiviral RNA silencing may be determined by the accessibility of target sites (Simon-Mateo and Garcia, 2006). More research should be done on these aspects to make more advancement, which would give us more insight into vsiRNA-mediated antiviral defense through PTGS.

### VsiRNAs Function to Downregulate Viral DNAs

Virus-derived small interfering RNAs can also be recruited by AGO proteins to form antiviral RISC that target viral DNA. It has been reported in plants that DNA methylation not only stabilizes transposons in the genome but is also involved in defense against invasive viral DNA genomes guided by abundant vsiRNAs (Chellappan et al., 2004; Vanitharani et al., 2005; Moissiard and Voinnet, 2006). DNA methylation at gene promoters can be triggered by dsRNAs through the RNA-directed DNA methylation (RdDM) pathway and induces TGS. The RdDM pathway requires 24-nt siRNAs for *de novo* DNA methylation of cytosines in all sequence contexts and AGO4 to target transcripts (Chan et al., 2004; Xie et al., 2004; Henderson et al., 2006). However, there is little molecular evidence of a vsiRNA-mediated RdDM pathway in plant antiviral defense. Studies on vsiRNA-mediated RdDM pathway have been conducted based on ssDNA plant *geminivirus* (Brough et al., 1992; Ermak et al., 1993; Wang et al., 2003, 2005; Raja et al., 2008). A group has shown that *Arabidopsis* plants defective in methyltrasferase or related cofactor activity are hypersensitive to geminivirus infection, suggesting that the viral genome is targeted by RdDM. They also demonstrated that Beet curly top virus L2-mutant DNA present in tissues that have recovered from infection is hypermethylated and that host recovery requires AGO4, a component of RdDM pathway (Raja et al., 2008). Recently, the same group reported that *Arabidopsis* Double-stranded RNA Binding 3 (DRB3) functions with DCL3 and AGO4 to induce repressive viral genome methylation against *geminiviruses* (Raja et al., 2014). This work demonstrates that the vsiRNA-mediated RdDM pathway is likely the predominant defense approach for hosts to counter plant DNA viruses during infection. Furthermore, we hypothesized that the vsiRNA-mediated RdDM pathway also requires various host factors such as reiterated GW/WG repeats, a conserved effector motif on the plant-specific DNA-directed RNA polymerase IVb (PolIVb), also called the AGO hook. As previous studies described, they are binding platforms for AGO4 and are essential for the biogenesis of 24-nt siRNAs (**Figure 1**; El-Shami et al., 2007).

### VsiRNAs Function to Downregulate Host Transcripts

Some have hypothesized that viruses might use sRNAs to silence specific host genes when there is near perfect complementarity (Addo-Quaye et al., 2008). However, the potential role of viral siRNAs in regulating host gene expression has rarely been reported. This idea can be first exemplified by the disease symptoms induced by the CMV Y-satellite RNA (Y-Sat), which relies entirely upon its helper virus (CMV) for replication and movement. In one instance, viral siRNA derived from the CMV-Y satellite RNA (Y-Sat) can specifically target the 22-nt sequence in *CHLI* mRNA, encoding a magnesium chelatase subunit. Down-regulation *CHLI* leads to impairment in the chlorophyll biosynthesis pathway and the resulting yellowing symptom in the leaves (Shimura et al., 2011). In another case, two *Peach latent mosaic viroid* (PLMVd)-derived siRNAs specifically target host *CHLOROPLASTIC HEAT-SHOCK PROTEIN 90* transcripts, resulting in the albino phenotype characteristic of peach leaves infected with PLMVd (Navarro et al., 2012). Recently, by performing artificial microRNA experiments in a transient expression system and by using RNA ligase-mediated rapid amplification of cDNA ends, a single small RNA derived from the virulence modulating region of two *Potato spindle tuber viroid* (PSTVd) variants was characterized. It targets several callose synthase genes of tomato plants (*CalS11*-like and *CalS12*-like), which are essential for callose formation during pathogen infection (Adkar-Purushothama et al., 2015). Intriguingly, we also identified a sRNA derived from a rice virus with the potential to target rice endogenous transcripts and induce abnormal phenotypes in rice (unpublished). These observations indicate vsiRNAs possess an ability to silence specific host genes with nearly perfect complementary. Nonetheless, according to results from degradome sequencing datasets from virus-infected plants and/or 5′ Rapid Amplification of cDNA Ends analysis of putative transcripts show that only rare endogenous genes were targeted by characterized siRNAs (Li et al., 2013; Miozzi et al., 2013), suggesting that vsiRNAs only occasionally regulate the host gene expression through PTGS.

### Suppression of vsiRNA Function by Various VSRs

Current research has verified the functions of vsiRNAs in targeting viral RNAs or DNAs and, occasionally, host transcripts. In turn, many plant viruses evolved VSRs counteract the antiviral silencing process mostly through arresting the functional vsiRNAs-RISC assembly (Csorba et al., 2015). Co-immunoprecipitation and small RNA profiling revealed that *Potyvirus* HC-Pro can associate with vsiRNAs during TuMV infection, suggesting that HC-Pro may interfere with vsiRNAs function in antiviral defense by binding vsiRNAs and blocking vsiRNA-RISC assembly (Garcia-Ruiz et al., 2015). P25 can inhibit the antiviral RNA silencing pathway by precluding AGO proteins from accessing viral RNA, as well as by directly inhibiting the formation of RNA silencing machinery (Brosseau and Moffett, 2015). *Polerovirus* P0 interacts with E3-ligase S-phase kinase regulated protein 1 (SKP1) through its F-box motif to enhance the degradation of multiple AGOs (AGO1, 2, 4–6, 9) before holo-RISC assembly. ToRSV suppressor coat protein (CP) binds to AGO1 to suppress its translational inhibitory activity and to enhance AGO1 degradation through autophagy (Karran and Sanfacon, 2014). RNase 3, a VSR encoded by *Sweet potato chlorotic stunt crinivirus* (SPCSV), affects vsiRNA function by using a completely different strategy, in which RNase3 cleaves 21–24-nt vsiRNAs into 14 bp products and renders them inactive, thus effectively preventing the formation of antiviral RISC (Kreuze et al., 2005; Cuellar et al., 2009). C2 protein, encoded by DNA virus *Beet severe curly top virus*, is an effector that counteracts antiviral defense by interfering with gene silencing and metabolic defense responses. C2 mediates a decrease in DNA methylation, which is linked with reduced accumulation of siRNAs derived from the methylated promoters, to result in upregulation of the corresponding coding genes (Yang et al., 2013; **Figure 1**). Regrettably, the activity of VSRs encoded by rice viruses in suppression of vsiRNAs function is completely unknown.

Though many evidences indicate that vsiRNAs play a key role in plant antiviral RNA silencing, the molecular mechanisms are elusive. Much work on the studies of biological roles of specific vsiRNAs and function mechanism of VSRs should be done in the future to further extend our understanding on the vsiRNAs function.

## The Application of Virus-Derived Small RNAs

### Use of vsiRNAs to Induce Antiviral Resistance

Current knowledge suggest RNA silencing make a great contribution to the resistance against pathogens on their respective crops (Nicaise, 2014). Whereas, naturally occurred vsiRNAs or miRNAs induced virus resistance is not enough for protecting the host plants from viral infection. By using hairpin constructs, dsRNA can be expressed in plants relatively easily, and RNA silencing is activated to silence the expression of genes of invading pathogens (Mansoor et al., 2006; Prins et al., 2008; Collinge et al., 2010). For viruses, this strategy is increasingly being used. Some cases directly characterize the application of vsiRNA in inducing antiviral resistance such as AsatsiR-12 (Zhu et al., 2011). However, many evidences indicating the application of vsiRNAs to induce antiviral resistance are indirect. In transgenic tobacco expressing hairpin RNA derived from TMV movement protein (MP) or CMV replication protein, two T4 transgenic lines with single copy were completely resistant to the corresponding virus (Hu et al., 2011). In rice, strong resistance is induced in transgenic rice plants expressing hairpin RNA of the viral genes, such as *RDV* viroplasm matrix protein Pns12 (Shimizu et al., 2009), *RSV* CP (Park et al., 2012), *Rice gall dwarf virus* Pns9 (Shimizu et al., 2012), *Rice grassy stunt virus* CP or MP (Shimizu et al., 2013). In transgenic maize, expressing the hairpin structure transcribed from the *CP* gene of *Sugarcane mosaic virus* (SCMV) led to inhibition of SCMV infection, although to varying degrees (Gan et al., 2014). Thus, in a variety of crops, based on the knowledge of vsiRNAs-mediated antiviral RNA silencing, expressing hairpin constructs strategy has been a potential approach with high efficiency to combat the virus.

### Use of vsiRNAs to Assemble Virus and Viroid Genomes

Additionally, the complete genome of a certain virus can be reconstructed based on vsiRNAs derived from the virus by combining small RNA deep sequencing with *de novo* assembly of viral siRNAs using bioinformatics tools. For instance, Ruiz-Ruiz et al. (2011) used vsiRNAs sequencing to reconstruct the full genome of the T318A Spanish *Citrus tristeza virus* (CTV) isolate that infects sweet (*Citrus sinensis* Osbeck) and sour orange (*C. aurantium* L.) and Mexican lime (*C. aurantifolia* Christ.) seedlings. Seguin et al. (2014) reconstructed DNA viruses from *Caulimoviridae* and *Geminiviridae* families by utilizing this approach, subsequently, they also reconstructed an emerging DNA virus and two viroids associated with economically important red blotch disease of grapevine to verify that vsiRNA-based deep sequencing allows for *de novo* reconstruction of any DNA or RNA virus genome and its microvariants (Seguin et al., 2014). A new virus, Citrus vein enation virus (CVEV), was identified as the causal agent of citrus vein enation disease by deep sequencing sRNAs from infected and healthy Etrog citron plants (Vives et al., 2013). Another research group reported the presence of a *Tobamovirus* on *Cicer arietinum* in Europe for the first time and a viroid referring to Hop stunt viroid (NC_001351.1) was reported in chickpea based on short RNAs sequencing (Pirovano et al., 2014).

### Use of vsiRNAs to Study Viral Population Genetics

Virus-derived small interfering RNAs-based deep sequencing was also used to survey the *in planta* virus population for the first time. Viral RNAs and vsiRNAs both congruently portrayed the mutational landscape of the virus within the plant host (Kutnjak et al., 2015). Next generation sequencing (NGS) technology has previously been shown to be a powerful tool for studying viral ecology (Stobbe and Roossinck, 2014) and viral populations (Beerenwinkel and Zagordi, 2011; Palmer et al., 2014; Chateigner et al., 2015; Skums et al., 2015). Nowadays, many population genetics experts prefer to study plant virus ecology or populations by sequencing the vsiRNAs. For instance, SG29 (aggressive) and Bau282 (mild), two representative isolates of a CTV population in Sicily, were sequenced from vsiRNAs of budlings of sweet orange grafted on sour orange trees. The phylogenetic relationships with Mediterranean and exotic isolates revealed that SG29 clustered within the “VT-Asian” subtype, whereas Bau282 belonged to cluster T30 (Licciardello et al., 2015). Notably, although vsiRNAs-based NGS technology is a more effective way to study virus ecology and populations, there is relatively high error rate. Thus, several computational pipelines such as ViVan (Isakov et al., 2015), ViVaMBC (Verbist et al., 2015), and LayerCake (Correll et al., 2015), tools with different applications for analyzing viral deep sequencing data, have been developed.

In summary, much of what we know already and what we will uncover in the future regarding plant viruses is possible by the study of vsiRNAs. Previous studies have proven that it is not only a feasible tool to control virus in crop plants by artificially expressing vsiRNAs, but also a high-throughput and cost-effective approach for reconstructing viral genomes, discovering novel virus, and studying viral ecology and populations by vsiRNAs deep sequencing experiments.

## Future Challenges

With the advance of NGS technologies, it is predicted that more studies using RNA profiling of plants infected with various pathogens will help identify more vsiRNAs. However, understanding the mechanism of action of vsiRNA-mediated antiviral silencing is a big challenge for researchers. Additionally, the interactions between host plants, viruses, and the abundant various vsiRNAs are extremely complex. The question of how to determine and classify the roles of a large number of vsiRNAs in the co-evolutionary battle between hosts and viruses also remains largely unanswered. Furthermore, it is unknown whether it is a general phenomenon that the vsiRNAs are able to induce disease by targeting host endogenous genes. Until now it is unknown whether vsiRNA-mediated endogenous gene silencing is a common mechanism in virus-infected plants. Albeit great advances have been made in our knowledge of sRNA-mediated plant immunity against viruses, studies on effective antiviral drugs against plant viruses and/or approaches on improving host resistance to virus disease are still deficient. Lastly, evidence suggests a potential method for controlling viral diseases is the expression of artificial sRNAs targeting the viral genome. However, any potential risks or safety issues of using this tool in crops must be evaluated.

## Conflict of Interest Statement

The authors declare that the research was conducted in the absence of any commercial or financial relationships that could be construed as a potential conflict of interest.
